# Clinical characteristics and treatment outcome of oropharyngeal squamous cell carcinoma in an endemic betel quid region

**DOI:** 10.1038/s41598-019-57177-1

**Published:** 2020-01-16

**Authors:** Tseng-Cheng Chen, Chen-Tu Wu, Jenq-Yuh Ko, Tsung-Lin Yang, Pei-Jen Lou, Cheng-Ping Wang, Yih-Leong Chang

**Affiliations:** 1 Department of Otolaryngology, National Taiwan University Hospital and National Taiwan University College of Medicine, Taipei, 10002 Taiwan; 2 Department of Pathology, National Taiwan University Hospital, National Taiwan University Cancer Center and National Taiwan University College of Medicine, Taipei, 10002 Taiwan; 3 Graduate Institute of Pathology, National Taiwan University College of Medicine, Taipei, 10002 Taiwan

**Keywords:** Head and neck cancer, Head and neck cancer

## Abstract

The clinical characteristics of oropharyngeal squamous cell carcinoma (OPSCC) may be different between endemic and non-endemic regions of betel nut chewing. The impact of combined alcohol drinking/betel quid chewing/cigarette smoking (ABC) exposure on the survival of OPSCC remains unclear. We reviewed the medical records of OPSCC patients between 1999 and 2013. Immunohistochemical staining of p16 and HPV genotype detection by DNA Polymerase chain reaction were both performed for each tumor. A total of 300 eligible patients including 74 HPV+ OPSCC patients and 226 HPV− OPSCC patients were enrolled. The 5-year disease-free survival rates for the HPV−, HPV+ OPSCC with and without ABC patients were 49.8%, 58.4% and 94%, respectively. The 5-year overall survival rates for the patients with HPV−, HPV+ OPSCC with and without ABC patients were 46%, 57.4% and 86%, respectively. Advanced locoregionally disease (T3/T4, N2/N3), HPV- OPSCC, combined 2 or all ABC exposure were the independent adverse prognostic factors for disease-free and overall survival. Therefore, our data suggest that in an endemic region of betel quid chewing, HPV− OPSCC comprises the majority of OPSCC and has a worse survival. Combined 2 or all ABC exposure had a significant negative impact on disease-free and overall survival.

## Introduction

Human papillomavirus (HPV) has been shown to be one of the causes of oropharyngeal squamous cell carcinoma (OPSCC), especially in patients without traditional risk factor exposure^1,2^. The virus contains two oncogenes, E6 and E7, which inactivates p53 and retinoblastoma, respectively^3^. Both pathways are involved in the carcinogenesis of HPV+ OPSCC. Whole exome sequencing data have demonstrated that HPV+ OPSCC has different genetic alterations from HPV− OPSCC, which is mostly caused by cigarette smoking^4,5^. In comparison with HPV− OPSCC, HPV+ OPSCC has better treatment response to chemoradiotherapy (CRT) and has a better survival^6^. The 8^th^ American Joint Committee on Cancer (AJCC) staging system downgrades HPV+ OPSCC staging and clinical trials on de-intensified treatment in HPV+ OPSCC are ongoing right now^7^.

In developed Western countries, HPV+ OPSCC is more common than HPV− OPSCC nowadays^8^. In the United States, HPV+ OPSCC accounts for more than 70% of all OPSCC^9^. In Taiwan, HPV− OPSCC is still more common than HPV+ OPSCC^10^. The reason for a higher frequency of HPV- OPSCC in Taiwan is due to betel quid chewing and cigarette smoking still being common. The prevalence of current betel quid chewer and cigarette smoker among men in Taiwan are about 10% and 30%, respectively^11,12^. Moreover, most of the patients with HPV− OPSCC and many patients with HPV+ OPSCC in Taiwan also have two or all exposures of alcohol drinking, betel quid chewing and cigarette smoking (ABC), which all are the risk factors strongly associated with OPSCC^13^. Therefore, clinical characteristics and treatment outcome of OPSCC in Taiwan may be different from those in other populations with only single or two exposures of alcohol drinking and cigarette smoking, without betel quid chewing. The aims of this study are to show the clinical characteristics of OPSCC in an endemic region of betel quid chewing and to analyse the impacts of ABC exposure on the survival of the OPSCC patients.

## Results

### Patient demographics

A total of 300 eligible patients diagnosed with OPSCC, including 74 (25%) patients with HPV+ OPSCC and 226 (75%) patients with HPV− OPSCC, were enrolled in this study. The mean age of all OPSCC patients included in our series was 54 ± 10 years (range, 29–83 years). The mean age of the HPV− OPSCC patients, HPV + OPSCC without and with ABC exposure patients were 53 ± 10 years (range, 29–80 years), 56 ± 12 years (range, 29–82 years) and 57 ± 11 years (range, 37–83 years), respectively (p = 0.15). The prevalence of HPV+ OPSCC after 2004 was higher than that before 2004. Among 226 patients with HPV− OPSCC, 191 (85%) patients had ABC exposure. Among 74 patients with HPV+ OPSCC, 38 (51%) patients had and the other 36 (49%) patients did not have ABC exposure. For all OPSCC patients, it was highly associated between alcohol drinking and betel quid chewing (r = 0.64), between betel quid chewing and cigarette smoking (r = 0.55) and between alcohol drinking and cigarette smoking (r = 0.69).

The clinicopathological characteristics are listed in Table 1. Female patients were significantly more common in the group of HPV+ OPSCC without ABC exposure than the groups of HPV− OPSCC and HPV+ OPSCC with ABC exposure (p = 0.001). HPV+ OPSCC had advanced nodal disease significantly more common than HPV− OPSCC (p = 0.02). Two hundred (89%) of 226 HPV− OPSCC were p16 negative and 206 (91%) of HPV− OPSCC were HPV DNA PCR negative. Sixty (81%) of 74 HPV+ OPSCC contained HPV subtype 16 and the other 14 (19%) tumor contained other types or multiple infections of high-risk HPV (subtypes 33, 35, 56, 58, 68).

**Table 1:** The clinicopathological characteristics of oropharyngeal squamous cell carcinoma.

Characteristics	HPV− OPSCC (n = 226)	HPV+ OPSCC with ABC exposure (n = 38)	HPV+ OPSCC without ABC exposure (n = 36)	P value
**Age (years)**				
Mean ± Standard deviation (range)	53 ± 10 (29~80)	57 ± 11 (37~83)	56 ± 12 (29~82)	0.15*
>50	138 (61.06%)	26 (68.42%)	24 (66.67%)	0.61
≦50	88 (38.94%)	12 (31.58%)	12 (33.33%)	
Gender				
Male	210 (92.92%)	37 (97.37%)	26 (72.22%)	0.001**
Female	16 (7.08%)	1 (2.63%)	10 (27.78%)	
Primary Tumor				
Tonsil	131 (57.96%)	28 (73.68%)	27 (75%)	0.07*
Tongue base	74 (32.74%)	7 (18.42%)	6 (16.67%)	
Soft palate	18 (7.96%)	3 (7.89%)	1 (2.78%)	
Multifocal	3 (1.33%)	0	2 (5.56%)	
T classification				
T3, T4	102 (45.13%)	18 (47.37%)	10 (27.78%)	0.13
T1, T2	124 (54.87%)	20 (52.63%)	26 (72.22%)	
N classification				
N2, N3	136 (60.18%)	27 (71.05%)	30 (83.33%)	0.02
N0, N1	90 (39.82%)	11 (28.95%)	6 (16.67%)	
Carcinogen exposure				
Alcohol	169 (74.79%)	24 (63.16%)	0	
Cigarette	185 (81.86%)	38 (100%)	0	
Betel quid	133 (58.85%)	31 (81.58%)	0	
Treatment				
Single modality (RT or OP)	15 (6.64%)	5 (13.16%)	0	0.007**
Two-modality	141 (62.39%)	28 (73.68%)	19 (52.78%)	
Three-modality	70 (30.97%)	5 (13.16%)	17 (47.22%)	
Tumor p16 status				
≥70%	26 (11.50%)	38 (100%)	36 (100%)	<0.001
<70%	200 (88.50%)	0	0	
Tumor HPV Genotype				
Type 16	15 (6.64%)	31 (81.58%)	29 (80.56%)	<0.001**
Others (Type 33,35,56,58,68)	4 (1.77%)	6 (15.79%)	5 (13.89%)	
Multiple (≥2, Type 16+)	1 (0.44%)	1 (2.63%)	2 (5.56%)	
Negative	206 (91.15%)	0	0	
Disease failure pattern				
Local recurrence	61 (26.99%)	5 (13.16%)	2 (5.56%)	0.004
Regional recurrence	36 (15.93%)	4 (10.53%)	0	0.013**
Distant metastasis	38 (16.81%)	8 (21.05%)	1 (2.78%)	0.07
Second primary malignancy	46 (20.35%)	3 (7.89%)	3 (8.33%)	0.05
Calendar year at diagnosis				
1999–2003	64 (28.32%)	3 (7.89%)	3 (8.33%)	0.003
2004–2008	86 (38.05%)	14 (36.84%)	19 (52.78%)	
2009–2013	76 (33.63%)	21 (55.26%)	14 (38.89%)	

Abbreviation: HPV, human papillomavirus; HPV−, HPV-negative; HPV+, HPV-positive; OPSCC, oropharyngeal squamous cell carcinoma; RT, radiotherapy; OP, curative operation alone; *using one-way ANOVA test; **using Fisher’s exact test.

### Survival outcome of OPSCC patients based on different HPV and ABC status

**Figure 1:**
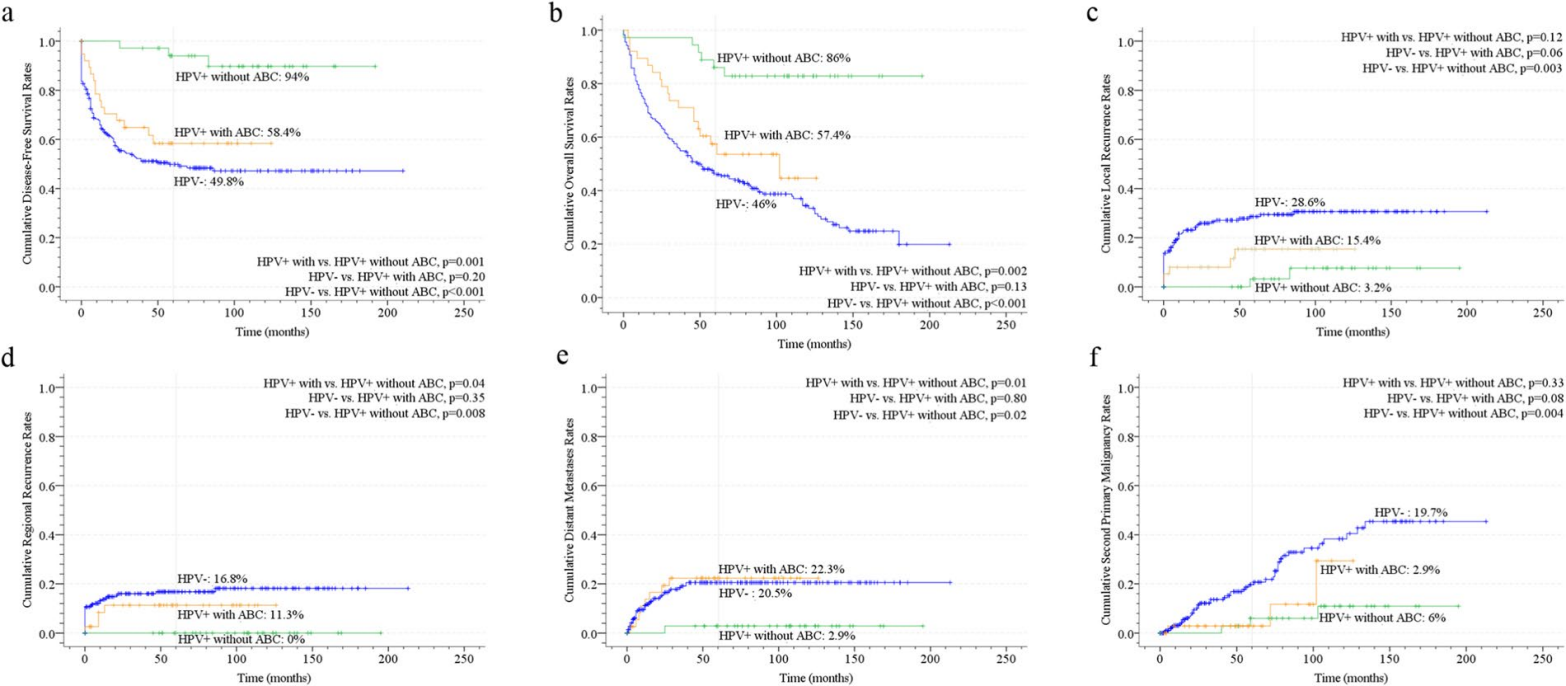
The survival outcome and disease control of human papillomavirus (HPV)− and HPV+ oropharyngeal squamous cell carcinoma (OPSCC) with and without alcohol, betel quid and cigarette smoking (ABC) in our series: (**a**) disease-free survival curves; (**b**) overall survival curves; (**c**) local recurrence rates; (**d**) regional neck recurrence rates; (**e**) distant metastases rates; (**f**) second primary malignancies rates.

The follow-up period was from 1 to 213 months, with a mean of 63 ± 50 months. The mean follow-up periods of the patients with HPV+ and HPV− OPSCC were 78 ± 42 and 58 ± 51 months, respectively. The time to disease progression was from 1 to 210 months, with a mean of 50 ± 50 months. The 5-year disease-free and overall survival rates of all patients were 48.3% and 52.3%, respectively. The 5-year disease-free survival rates of the patients with HPV− OPSCC, HPV+ OPSCC with and without ABC exposure were 49.8%, 58.4% and 94%, respectively (Fig. 1a). After adjusting with major competing risk factor (death) by cumulative incidence competing risk (CICR) method, the adjusted 5-year disease-free survival rates of the patients with HPV− OPSCC, HPV+ OPSCC with and without ABC exposure were 52.7%, 60.5% and 94.4%, respectively. The 5-year overall survival rates of the patients with HPV− OPSCC, HPV+ OPSCC with and without ABC exposure were 46%, 57.4% and 86%, respectively (Fig. 1b). The disease-free and overall survival between HPV− OPSCC and HPV+ OPSCC with ABC exposure were not significantly different (p = 0.2, p = 0.13, respectively), but there were significant differences in both disease-free and overall survival between HPV+ OPSCC with and without ABC exposure (p = 0.001, p = 0.002, respectively). The 5-year local recurrence rates of the patients with HPV− OPSCC, HPV+ OPSCC with and without ABC exposure were 28.6%, 15.4% and 3.2%, respectively (Fig. 1c). After adjusting with major competing risk factor (death) by CICR method, the adjusted 5-year local recurrence rates of the patients with HPV− OPSCC, HPV+ OPSCC with and without ABC exposure were 26.2%, 13.2% and 2.87%, respectively. The 5-year regional recurrence rates of the patients with HPV− OPSCC, HPV+ OPSCC with and without ABC exposure were 16.8%, 11.3% and 0%, respectively (Fig. 1d). After adjusting with major competing risk factor (death) by CICR method, the adjusted 5-year regional recurrence rates of the patients with HPV− OPSCC, HPV+ OPSCC with and without ABC exposure were 15.5%, 10.5% and 0%, respectively. The 5-year distant-metastasis rates of the patients with HPV− OPSCC, HPV+ OPSCC with and without ABC exposure were 20.5%, 22.3% and 2.9%, respectively (Fig. 1e). After adjusting with major competing risk factor (death) by CICR method, the adjusted 5-year distant-metastasis rates of the patients with HPV− OPSCC, HPV+ OPSCC with and without ABC exposure were 16.8%, 21.1% and 2.78%, respectively. The 5-year cumulative rates of the metachronous second primary malignancy of the patients with HPV− OPSCC, HPV+ OPSCC with and without ABC exposure were 19.7%, 2.9% and 6.0%, respectively, but the 10-year cumulative rates of the metachronous second primary malignancy of those groups were 38.3%, 29.4% and 10.9%, respectively (Fig. 1f). After adjusting with major competing risk factor (death) by CICR method, the adjusted 5-year cumulative rates of the metachronous second primary malignancy of the patients with HPV− OPSCC, HPV+ OPSCC with and without ABC exposure were 12.6%, 2.63% and 5.64%, respectively.

### Risk stratification for survival outcomes of the OPSCC patients

The univariate analyses of all possible factors on survival are shown in Table 2. Male gender (p = 0.001), locally advanced T3/T4 tumor (p < 0.001), negative p16 staining (p < 0.001), negative HPV DNA PCR (p < 0.001), alcohol drinking (p < 0.001), betel quid chewing (p < 0.001) and cigarette smoking (p < 0.001) were the adverse prognostic factors for disease-free survival. Male gender (p = 0.001), locally advanced T3/T4 tumor (p < 0.001), negative p16 staining (p < 0.001), negative HPV DNA PCR (p < 0.001), alcohol drinking (p < 0.001), betel quid chewing (p < 0.001) and cigarette smoking (p < 0.001) were the adverse prognostic factors for overall survival.

**Table 2:** Univariate analysis of the prognostic factors for survival.

Factors	5-year Disease-Free Survival	P value	5-year Overall Survival	P value
Age (years)				
>50	58.4%	0.41	51.8%	0.46
≤50	53.4%		53.2%	
Gender				
Male	52.9%	0.001	49.4%	0.001
Female	88.9%		81.3%	
Primary Tumor				
Tonsil	58.6%	0.22	52.4%	0.68
Tongue Base	50.1%		51.6%	
Soft Palate	53.1%		55.0%	
Multifocal	0%		40.0%	
T classification				
T3/T4	41.8%	<0.001	37.4%	<0.001
T1/T2	67.4%		63.6%	
N classification				
N2/N3	53.0%	0.09	50.1%	0.14
N0/N1	62.5%		56.0%	
Tumor p16 status				
Negative	47.5%	<0.001	44.0%	<0.001
Positive	73.7%		68.7%	
HPV DNA PCR status				
Negative	47.2%	<0.001	43.7%	<0.001
Positive	75.6%		71.0%	
Treatment				
Single modality (RT or OP)	50.3%	0.26	57.0%	0.54
Two-modality	54.1%		48.0%	
Three-modality	62.5%		59.7%	
Alcohol drinking				
Positive	43.1%	<0.001	39.8%	<0.001
Negative	78.1%		74.6%	
Betel quid chewing				
Positive	39.2%	<0.001	35.7%	<0.001
Negative	75.6%		71.9%	
Cigarette smoking				
Positive	46.2%	<0.001	42.5%	<0.001
Negative	83.9%		80.3%	
HPV and ABC exposure history				
HPV− OPSCC	49.8%	<0.001	46.0%	<0.001
HPV+ OPSCC with ABC	58.4%		57.4%	
HPV+ OPSCC without ABC	94%		86.0%	
HPV− OPSCC and ABC exposure				
Combined 2 or all exposure	40.5%	<0.001	37.5%	<0.001
Single exposure	66.2%		55.6%	
No exposure	82.9%		82.8%	
HPV+ OPSCC and ABC exposure				
Combined 2 or all exposure	55.7%	0.001	50.9%	<0.001
Single exposure	71.4%		85.7%	
No exposure	94%		86.0%	

Abbreviation: HPV, human papillomavirus; HPV−, HPV-negative; HPV+, HPV-positive; PCR, Polymerase chain reaction; IHC, immunohistochemistry; ABC. Alcohol drinking/Betel quid chewing/Cigarette smoking; OPSCC, oropharyngeal squamous cell carcinoma; RT, radiotherapy; OP, curative operation alone.

**Figure 2:**
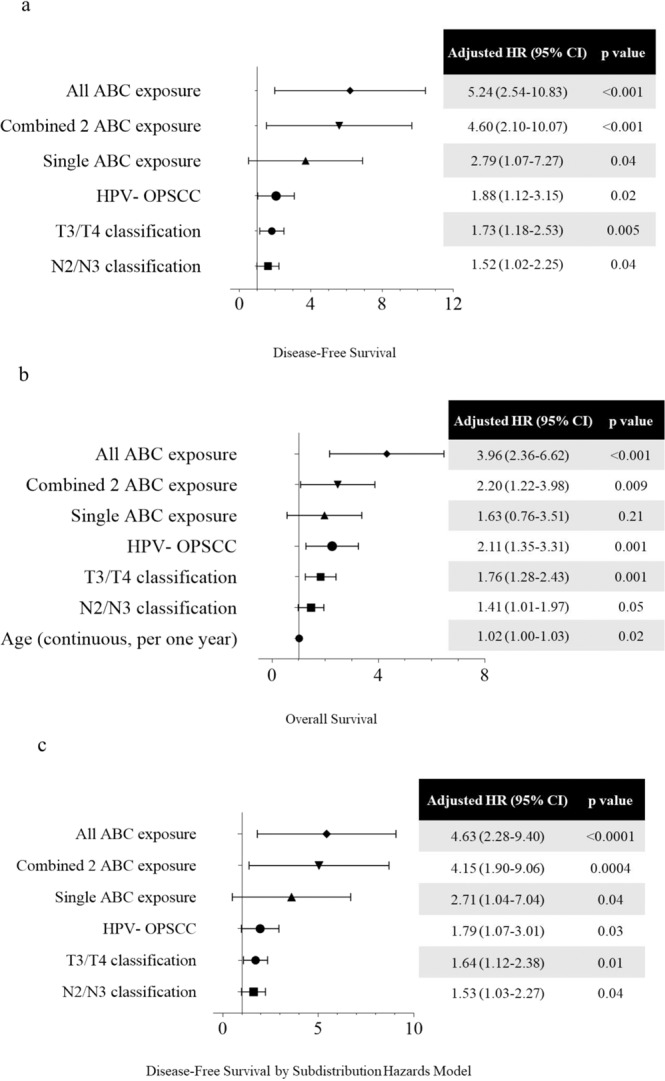
The multivariable analyses of all prognostic factors for survival using Cox proportional hazards model with a forward selection procedure (age work as continuous variable; all other factors work as categorical variable): (**a**) disease-free survival; (**b**) overall survival; (**c**) The adjusted multivariant analyses with competing risk factor, death, by Subdistribution hazards model for disease-free survival (Abbreviation: HR: hazard ratio; ABC, Alcohol drinking/Betel quid chewing/Cigarette smoking; OPSCC, oropharyngeal squamous cell carcinoma; HPV, human papillomavirus; HPV−, HPV-negative).

**Figure 3:**
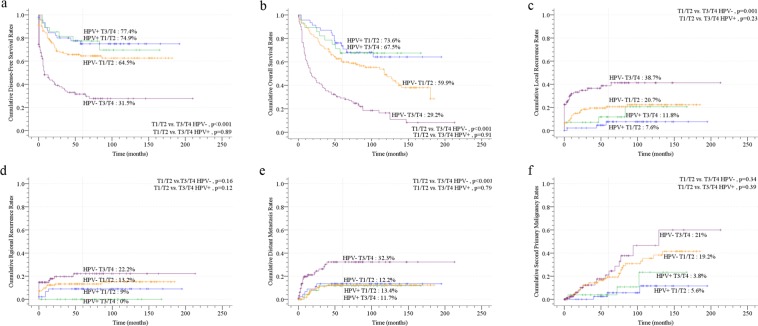
The survival and disease control outcomes of T1/T2 and T3/T4 human papillomavirus (HPV)− and T1/T2 and T3/T4 HPV+ oropharyngeal squamous cell carcinoma (OPSCC) in our series: (**a**) disease-free survival curves; (**b**) overall survival curves; (**c**) local recurrence rates; (**d**) regional neck recurrence rates; (**e**) distant metastases rates; (**f**) second primary malignancies rates.

The multivariant analyses by Cox Proportional Hazard Model for the disease-free and overall survivals are shown in Fig. 2a,b. After adjusting with major competing risk factor (death) by Subdistribution Hazard Model, the significant risk factors for disease-free survival are shown in Fig. 2c. Locoregionally advanced tumor (T3/T4 and N2/N3), combined 2 ABC exposure, all ABC exposure and HPV− OPSCC were the independent adverse factors for both disease-free and overall survival. The impacts of locally advanced T3/T4 tumor on disease control and survival stratified by HPV are shown in Fig. 3. In HPV+ OPSCC, T3/T4 tumor had similar survival and locoregional control to T1/T2 tumor. In HPV− OPSCC, T3/T4 tumor had significantly worse disease-free survival (p < 0.001, Fig. 3a), overall survival (p < 0.001, Fig. 3b), local control (p = 0.001, Fig. 3c) and distant failure (p < 0.001, Fig. 3e), than T1/T2 tumor.

The ABC exposure was also an independent adverse factor for disease-free survival. Multiple ABC exposure was the independent adverse factor for disease-free and overall survival. The patients with two or all ABC exposure had the worst disease-free and overall survival than those with single risk factor exposure, in both HPV− and HPV+ OPSCC. Among the patients with HPV− OPSCC, the 5-year disease-free survival rates of the patients with 2 or more ABC exposure, single ABC exposure and no exposure were 40.5%, 66.2% and 82.9%, respectively (p < 0.001), and the 5-year overall survival rates of the patients with 2 or more ABC exposure, single ABC exposure and no exposure were 37.5%, 55.6% and 82.8%, respectively (p < 0.001). Among the patients with HPV+ OPSCC, the 5-year disease-free survival rates of the patients with 2 or more ABC exposure, single ABC exposure and no exposure were 55.7%, 71.4% and 94%, respectively (p = 0.001), and the 5-year overall survival rates of the patients with 2 or more ABC exposure, single ABC exposure and no exposure were 50.9%, 85.7% and 86%, respectively (p < 0.001), which were all better than those with HPV− OPSCC, respectively (Table 2).

## Discussion

The HPV+ OPSCC is a subtype of OPSCC, which is associated with a better response to treatment and a better outcome. In western countries, where is betel quid non-endemic region, the HPV+ OPSCC patients tend to be younger, have a higher socioeconomic status and are less likely to smoke^6,8,9^. However, it has been demonstrated that each of ABC exposures is strongly associated with the risk for OPSCC^13,14^. Among them, the betel quid may be the most potent carcinogen associated with non-nasopharyngeal carcinoma head and neck cancer (HNC), including OPSCC^13,14^. Because most patients with OPSCC in Taiwan have two or all exposures of ABC, especially the betel quid, which is different from that in other populations with only alcohol or cigarette exposure, the clinical characteristics and survival of OPSCC in Taiwan may not be the same as those in non-endemic region of betel quid chewing.

Firstly, the incidence and prevalence trends of HPV− and HPV+ OPSCC were different between the non-endemic and endemic regions of betel quid chewing. The incidences of HPV− OPSCC in the non-endemic betel quid regions are decreasing^15,16^. On the contrary, the incidence of OPSCC is increasing because of the rapidly rising incidence of HPV+ OPSCC, which accounts for more than 70–90% of all OPSCC^8,9,16^. In an endemic region of betel quid chewing, HPV− OPSCC still accounts for the majority of OPSCC^17^. Our previous population-based studies showed that the incidences of all HNC associated with ABC including OPSCC in Taiwan are still increasing to date and only about one-third of OPSCCs were HPV+ OPSCC^10,18^. The incidence of HPV+ OPSCC is really rising in the recent years in Taiwan, similar to other developed countries because of the changes of sexual behaviour among younger age cohorts, but HPV− OPSCC is still more common than HPV+ OPSCC and the incidence of HPV− OPSCC is also increasing with a similar rate^18^.

Secondly, it has been shown that HPV+ OPSCC has significantly less locoregional recurrence and distant failure so that the survival of HPV+ OPSCC is much better than that of HPV− OPSCC^6,19,20^. This study showed the same finding that HPV+ OPSCC, especially without ABC exposure, also has a significantly better treatment response, less recurrence and a better survival in an endemic region of betel quid chewing. However, because the majority of the OPSCC tumor was HPV−, the overall survival in an endemic region of betel quid chewing was poorer than that in non-endemic region of betel quid chewing. The overall survival of OPSCC in this study (5-year overall survival: 52.3%) was still lower than that of the SEER database in USA (5-year overall survival: 65.8%)^21^ although the treatment outcome of other HNCs in Taiwan, for example, oral cavity cancer, is comparative to that in the developed countries^22,23^.

Furthermore, it has been shown that cigarette smoking worsens the survival of the patients with HPV+ OPSCC in Western countries and the patients with HPV+ OPSCC and cigarette smoking is classified as an intermediate risk group in terms of treatment outcome^6,24^. In an endemic region of betel quid chewing, the majority of patients with HPV− OPSCC and about half of the patients with HPV+ OPSCC chewed betel quid in addition to alcohol drinking and cigarette smoking. This study showed that ABC exposure had a significantly worse disease-free and overall survival for all OPSCC patients, either the patients with HPV− OPSCC or the patients with HPV+ OPSCC. However, it is difficult to clarify the impact of each risk factor exposure, especially the betel quid, on treatment outcome because the majority of the patients had two or all ABC exposures, especially betel quid chewers. Therefore, we stratified the patients by single or combined two or all ABC exposures in this study, which is easier and more clinically relevant. Combined two or all ABC exposure had the independent worst effect on disease-free and overall survival of OPSCC patients. The patients with HPV+ OPSCC and combined two or all ABC exposures had a significantly poorer prognosis than those with single exposure, and so did the patients with HPV− OPSCC. The 5-year disease-free and overall survival rates of the patients with HPV+ OPSCC and 2 or more ABC exposures were 55.7% and 50.9%, which were as poor as those with all HPV− OPSCCs (49.8%, 46%, respectively). Therefore, the negative impact of traditional carcinogen exposure, especially combined exposure, on the survival of the patient should be considered in terms of prognosis although this study still agrees with down-staging of HPV+ OPSCC in the American Joint Committee on Cancer 8th Edition Cancer Staging system^7^ because of no differences of local control and survival between T3/4 and T1/2 HPV+ OPSCC^2,20^. In an endemic region of betel quid chewing, not only cigarette but also betel quid and alcohol should be considered as the adverse prognostic factors because the patients who had two or all ABC exposures had the worst prognosis, even with HPV+ OPSCC. For the clinical trials on the de-intensified treatment for HPV+ OPSCC, it may be necessary to take combined ABC exposure, not only cigarette smoking, into account in allocating the patients. For choice of treatment in clinical practice, the patients with HPV+ OPSCC and two or more ABC exposures may be not a good candidate for the de-intensified treatment approach.

Second primary malignancy also causes patients’ death^25^. HPV− OPSCC has a significantly higher risk for metachronous second primary malignancy, which further lowers the survival, than HPV+ OPSCC, because the patients with HPV− OPSCC usually have carcinogen exposure, which causes field cancerization on the upper aerodigestive tract^26^, but HPV infection doesn’t cause obvious field cancerization^27^. Especially in Taiwan, most patients have multiple ABC exposures so that the prevalence and the cumulative incidence of second primary malignancy may be higher than those in other countries where are not endemic regions of ABC^25,28,29^. In this study, about 20% of patients with HPV− OPSCC had metachronous second primary malignancy, much higher than 8% of patients with HPV+ OPSCC. Therefore, the follow-up strategy for HPV− OPSCC should be different and more aggressive than that of HPV+ OPSCC in order to improve the survival by early diagnosis of the second primary malignancy in addition to recurrent diseases. Regular screening of synchronous/metachronous second primary malignancy should be considered in patients with OPSCC in an endemic region of betel quid chewing, especially for esophageal cancer, which is not uncommon and is the most lethal and difficult to diagnose at early stage without endoscopy screening^28^. Nowadays, endoscopic screening for second primary esophageal cancer is suggested for patients with pharyngeal cancers at diagnosis or during the follow-up after treatment in most hospitals in Taiwan, trying to improve the survival^28,30^.

There are some limitations in our series. First, with regard to the dose of ABC consumption, it is a little difficult to quantify the dose of alcohol/betel quid/cigarette exposure for every patient is this retrospective study. Therefore, it is really difficult to calculate the dose-relationship between the risk and the alcohol/betel quid/cigarette exposure from this cohort. Second, although the method we used to define HPV+ OPSCC, positive HPV− Polymerase chain reaction (PCR) sequencing and p16 immunohistochemistry (IHC) staining >70%, has been reported to have adequate sensitivity and specificity in detecting HPV-associated OPSCC^31^, there may be some discrepancies in the interpretation of our results in relation to real HPV-associated OPSCC.

In conclusion, in an endemic region of betel quid chewing, the majority of OPSCC patients still is HPV− OPSCC although the incidence of HPV+ OPSCC is also increasing. The treatment response and prognosis of HPV+ OPSCC without ABC exposure are good, which are similar to those in the non-endemic region of betel quid chewing. The locally advanced (T3/T4) tumor, HPV negative status and combined two or all ABC exposure were the independent adverse prognostic factors for disease-free and overall survival. HPV− OPSCC has a significantly higher risk for second primary malignancy.

## Methods

### Patient population

We retrospectively reviewed the medical records of the patients who were diagnosed with OPSCC and received curative-intended treatment at National Taiwan University Hospital between January 1999 and August 2013. Ethical approval for this study was obtained from institutional board at National Taiwan University Hospital (Approval Number: 201011033RC). Informed consent was obtained from all participants. All methods used in this study were performed by the relevant guidelines and regulations. The inclusion criteria were the OPSCC patients with corresponding Formalin-Fixed, Paraffin-Embedded tissue for IHC staining and HPV PCR analyses. The exclusion criteria included the patient with previously treated OPSCC, the patient with malignancies other than OPSCC, the patient with a simultaneous second primary cancer, and the patient with a previous history of radiotherapy involving the head or neck region due to other diseases. The TNM status of each tumor was classified according to the 2010 criteria of the AJCC^32^.

### Primary OPSCC p16 IHC staining

Primary tumor sections of 4-μm thickness were deparaffinized and pre-treated for antigen retrieval by autoclave heating (121 °C) in 10 mM sodium citrate buffer (pH 6.0) for 10 min. These sections were blocked for endogenous peroxidase activity with 3% H_2_O_2_ in methanol for 10 min and then washed in phosphate-buffered saline (PBS). Thereafter, the sections were immersed in UltraVision Protein Block (Thermo Fisher Scientific, Fremont, LA, USA) for 10 min, covered with a primary rabbit monoclonal antibody specific for p16 (clone: EP1215Y, Epitomics, Abcam Company, Burlingame, CA, USA) and incubated for one hour at room temperature. Immunoreactions were performed using UltraVision Quanto Detection System HRP DAB (Thermo Fisher Scientific, Fremont, LA, USA). Immunohistochemical evaluation of p16 in OPSCC specimens was based on the intensity and extent of nuclear and cytoplasmic reactivity. Positive p16 expression was defined as strong and diffuse nuclear and cytoplasmic staining in 70% or more of the tumor cells^6^. Two independent pathologists (Y-L.C. and C-T.W.) were involved in the assessment of tumor p16 expression.

### Primary OPSCC HPV PCR analysis

We used a commercial EasyChip® HPV blot kit (King Car, Taiwan) to carry out the HPV genotyping of the polymerase chain reaction (PCR) products of HPV and GAPDH. The quality of the HPV blot meets the requirement for class III GMP certification. The kit allows specific detection of 39 HPV genotypes (HPV types 6, 11, 16, 18, 26, 31, 32, 33, 35, 37, 39, 42, 43, 44, 45, 51, 52, 53, 54, 55, 56, 58, 59, 61, 62, 66, 67, 68, 69, 70, 72, 74, 82, CP8061, CP8304, L1AE5, MM4, MM7 and MM8, as well as three intrinsic controls) and is based on reverse hybridization. The detailed procedures of HPV genotype determination are as described in a previous work^33^.

### Statistical analysis

In this study, the tumor with both positive p16 staining (≥70%) and positive HPV DNA PCR test (detectable HPV genotype) was regarded as HPV+ OPSCC^34,35^. All statistical analyses were performed using the SPSS software package, version 16.0 (SPSS Inc., Chicago, IL). The one-way analysis of variance (ANOVA) was used to determine difference in age among the patients with HPV− tumor, HPV+ tumor without and with ABC exposure. Fisher’s exact tests and chi-square tests were used to determine differences in other clinical characteristics. The data of ABC exposure were obtained from medical and/or cancer registry record of our hospital. All patients with the history of daily exposure more than 6 months would be regarded as positive^13^. The correlation among ABC exposure was tested using the Phi coefficient. The starting point of the follow-up period was defined as the completion of the comprehensive treatment for each patient. The end point of the follow-up period was defined as the time when the patient expired, loss to follow-up, or June 2017. The primary outcomes were disease-free and overall survival. The secondary outcomes were local recurrence, regional recurrence and distant failure. The rates of disease-free survival, overall survival, local recurrence, regional recurrence and distant failure were calculated using the Kaplan-Meier product limit method. All sites of persistent, residual or recurrent tumor were recorded as treatment failures in terms of the disease-free survival; and all deaths were recorded against the overall survival parameter. Significance levels among the curves were determined using the log-rank test. The CICR method was used to adjust the major competing factor, death, for cumulative rates of disease-free survival, local recurrence, regional recurrence and distant failure. A multivariable Cox proportional hazards model with a forward selection procedure was used to estimate the effects of all possible covariates (Age work as continuous variable; all other factors work as categorical variable) on disease-free and overall survival endpoints. Finally, the Subdistribution hazard model was used to adjust the major competing risk factor, death, for disease-free survival. A p-value < 0.05 was considered statistically significant.
